# New Opportunities and Challenges of Early Psychosis

**DOI:** 10.3390/jcm11216531

**Published:** 2022-11-03

**Authors:** Marta Rapado-Castro

**Affiliations:** 1 Department of Child and Adolescent Psychiatry, Institute of Psychiatry and Mental Health, Hospital General Universitario Gregorio Marañón, School of Medicine, Universidad Complutense, IiSGM, CIBERSAM, ISCIII, 28009 Madrid, Spain; mrapado@iisgm.com; 2 Melbourne Neuropsychiatry Centre, Department of Psychiatry, The University of Melbourne and Melbourne Health, 161 Barry Street, Carlton South, VIC 3053, Australia

Over the past two decades, the early psychosis field has advanced and expanded substantially. A great deal of progress has been made in understanding phenomenology, neurobiology, and outcomes during the early phase of psychotic disorders. Although there has been extensive investigation of early signs of risk and evidence-based treatments, psychotic disorders continue to severely affect both young people and adults. Schizophrenia and other psychotic disorders represent the third leading cause of disability-adjusted life years worldwide [1]. Thus, the early diagnosis and treatment of first-episode psychosis (FEP) is crucial for short- and long-term prognosis. Delayed identification of symptoms and a longer duration of untreated psychosis may lead to challenges in functional and clinical outcomes. Effective and timely interventions have been shown to improve the psychopathology, quality of life, and vocational development of individuals with psychosis [2]. With major unmet needs such as cognitive negative symptoms and functional outcome, early psychosis research still faces several conceptual, methodological, and treatment challenges, some of which we review in this Special Issue of the *Journal of Clinical Medicine*. Top world-leading experts and research groups discuss their latest findings on prevalent issues, such as understanding neurobiology, course, and outcomes during the early phases of psychosis.

In this issue, in order to understand the process of recovery and what promotes or hinders it during this early critical period, the research by Molina-García et al. [3] and by Salagre et al. [4] focuses on exploring predictors and risk and resilient profiles for functional impairment. Both these authors present baseline and longitudinal data from the “Phenotype-genotype and environmental interaction; application of a predictive model in first psychotic episodes (PEPs study)” [5]. In addition, the study from Molina-García et al. [3] includes a sample for individuals up to 18 years old with a FEP from the “Children and adolescent first episode psychosis study (CAFEPS study)” [6]. It is very timely that both these authors are using a comprehensive statistical approach to the investigation of functional outcomes in PEP with the aim of identifying different trajectories of functional impairment in the 2 years after the FEP. In the study by Molina-García et al. [3], the authors report on four trajectories of psychosocial functioning in a sample of 255 FEP individuals (age range 10–36 years) according to the age of onset of the FEP and the premorbid intellectual functioning (pIQ) of the subjects. In particular, they found a subgroup of early onset with low pIQ that presented with poorer and persistent global psychosocial functioning at two-year follow-up. Conversely, they report on a subgroup of adult onset with average pIQ presenting with average functioning over time, with intermediate values for the early onset with average pIQ and adult onset with low pIQ subgroups [3]. Likewise, the study from Salagre et al. [4] found four trajectories of psychosocial functioning following FEP in a sample of 275 adult participants: two trajectories indicative of a persistent functional impairment and two describing a resilient course. In addition, these latter authors report on putative factors that might mediate functional resilience, such as better socioeconomic status and premorbid adjustment, milder negative symptoms, and more preserved cognitive functioning at the time of the FEP [4]. Taken together, findings from these two studies may have implications for the development of novel personalized treatment interventions directed to target functional impairments, calling for further investigation of protective and underlying mechanisms that support cognitive functioning as a crucial factor, particularly relevant for the functional outcome and prognosis in the early stages of psychosis.

Along these lines, the studies by Rapado-Castro et al. [7] and by Schermitzler et al. [8] examine neurobiological correlates [7] and risk factors for cognitive function and motivation [8] in psychosis. Rapado-Castro et al.’s [7] results suggest a particular association between decreased gray matter volume of the fronto-parietal lobes and a poorer working-memory performance over the first 2 years of psychosis in adolescents with a PEP, highlighting the role of the age of onset of the FEP in this relationship [7]. In particular, loss of gray matter volume in the frontal and parietal regions and younger age at the time of the FEP were associated with decreased working-memory function over time in a subsample (N = 33 adolescents, mean age 15.82) who had valid longitudinal cognitive and magnetic resonance imaging from the previously mentioned CAFEPS study [6]. Their results support the presence of a neurobiological substrate for cognitive impairment. Moreover, these authors report on a specific reduction in frontal left gray matter volume as a potential predictive biomarker underlying the observed working-memory impairment in this group. Importantly, these two events co-occur at a crucial time in adolescence particularly sensitive to working memory and frontal brain volume changes, which might explain the observed “disruption” in these neurodevelopmental processes in adolescents within the age range of the participants (11 to 17 years old) [7]. On the other hand, the study from and Schermitzler et al. [8] examines the neural mechanisms of nicotine dependence and psychosis in relationship to deficits in cognitive function and motivation in a large sample of individuals with FEP (N = 404) followed over two years within the Recovery After an Initial Schizophrenia Episode—Early Treatment Program (RAISE–ETP [9]). Tobacco use is highly prevalent and has been largely associated with cognitive function in FEP. The overlapping mechanisms driving nicotine dependence and psychosis have been described, as nicotine use stimulates both the cholinergic and dopaminergic systems, which are systems consistently linked to impairments in neurocognition and motivation and shown to be altered in psychosis. In this secondary analysis of the RAISE-ETP cohort, the authors report on a significant relationship between smoking status and motivation, but not global cognition in individuals experiencing FEP [8]. They found that non-smokers showed greater gains in motivation and had enhanced functional outcomes over two years of treatment, highlighting the importance of targeting motivation when working with the large proportion of smoking FEP individuals in our clinics. 

Another debilitating and common factor impacting functioning in FEP is antipsychotic-induced weight gain, an adverse side effect of antipsychotic treatment. In this vein, the study by Panizzutti et al. [10] in this issue examines the impact of differential gene expression in neuronal-like cells treated with antipsychotics (Pillinger P-score) on the propensity of antipsychotic drugs to cause weight gain. The authors demonstrated that commonly prescribed antipsychotic drugs can change the expression of genes involved in the lipid biosynthesis and metabolic pathways, and that SREBF transcription factors may play a role in these effects. In particular, “the effects of antipsychotic drugs on lipid metabolism may influence white matter structure (therapeutic effect) and the risk of weight gain, lipid disturbances, and, consequently, metabolic syndrome (adverse effects)” [10]. Understanding the underlying pathways of such risk factors and adverse effects of these drugs could inform a personalized medicine approach for treating individuals with psychosis.

Collectively, the findings reported in this Special Issue on “New Opportunities Additionally, Challenges Of Early Psychosis” in the *Journal of Clinical Medicine* provide evidence that may guide the development of new therapeutic approaches based on the neurodevelopmental processes (brain and functional/cognitive changes) occurring at the time of the first episode. Further understanding of the relationships between plausible biomarkers, resilience, risk factors, and long-term outcomes in people with FEP will help us develop better interventions that enhance functioning, as well as therapeutic strategies to aid in smoking cessation, managing the adverse effects of antipsychotic medication, and possibly preventing or delaying the onset of psychotic disorders and/or its long-term functional impairment. Moreover, all these studies demonstrate that an integrative approach is needed from the very early stages, motivating further exploration of the impact of potential risk and cognitive factors, with specific contributions to the process of recovery and functional outcomes in psychosis.

## References

[1] Gore F.M., Bloem P.J., Patton G.C., Ferguson J., Joseph V., Coffey C., Sawyer S.M., Mathers C.D. (2011). Global burden of disease in young people aged 10-24 years: A systematic analysis. Lancet.

[2] Fusar-Poli P., McGorry P.D., Kane J.M. (2017). Improving outcomes of first-episode psychosis: An overview. World Psychiatry.

[3] Molina-García M., Fraguas D., del Rey-Mejías Á., Mezquida G., Sánchez-Torres A.M., Amoretti S., Lobo A., González-Pinto A., Andreu-Bernabeu Á., Corripio I. (2021). The Role of Premorbid IQ and Age of Onset as Useful Predictors of Clinical, Functional Outcomes, and Recovery of Individuals with a First Episode of Psychosis. JCM.

[4] Salagre E., Grande I., Solé B., Mezquida G., Cuesta M.J., Díaz-Caneja C.M., Amoretti S., Lobo A., González-Pinto A., Moreno C.L. (2021). Exploring Risk and Resilient Profiles for Functional Impairment and Baseline Predictors in a 2-Year Follow-Up First-Episode Psychosis Cohort Using Latent Class Growth Analysis. JCM.

[5] Bernardo M., Bioque M., Parellada M., Saiz Ruiz J., Cuesta M.J., Llerena A., Sanjuán J., Castro-Fornieles J., Arango C., Cabrera B. (2013). Assessing clinical and functional outcomes in a gene-environment interaction study in first episode of psychosis (PEPs). Rev. Psiquiatr. Salud. Ment..

[6] Castro-Fornieles J., Parellada M., Gonzalez-Pinto A., Moreno D., Graell M., Baeza I., Otero S., Soutullo C.A., Crespo-Facorro B., Ruiz-Sancho A. (2007). The child and adolescent first-episode psychosis study (CAFEPS): Design and baseline results. Schizophr. Res..

[7] Rapado-Castro M., Villar-Arenzana M., Janssen J., Fraguas D., Bombin I., Castro-Fornieles J., Mayoral M., González-Pinto A., de la Serna E., Parellada M. (2021). Fronto-Parietal Gray Matter Volume Loss Is Associated with Decreased Working Memory Performance in Adolescents with a First Episode of Psychosis. JCM.

[8] Schermitzler B., Miley K., Vinogradov S., Ramsay I.S. (2021). Smoking Is Related to Reduced Motivation, But Not Global Cognition, in the First Two Years of Treatment for First Episode Psychosis. J. Clin. Med..

[9] Kane J.M., Robinson D.G., Schooler N.R., Mueser K.T., Penn D.L., Rosenheck R.A., Addington J., Brunette M.F., Correll C.U., Estroff S.E. (2016). Comprehensive Versus Usual Community Care for First-Episode Psychosis: 2-Year Outcomes From the NIMH RAISE Early Treatment Program. Am. J. Psychiatry.

[10] Panizzutti B., Bortolasci C.C., Spolding B., Kidnapillai S., Connor T., Richardson M.F., Truong T.T.T., Liu Z.S.J., Gray L., Kim J.H. (2021). Biological Mechanism(s) Underpinning the Association between Antipsychotic Drugs and Weight Gain. JCM.

